# Comparison of Core Decompression and Fibular Strut Graft in Early Avascular Necrosis of the Hip: Assessment of Postoperative Pain and Functional Outcomes

**DOI:** 10.7759/cureus.92733

**Published:** 2025-09-19

**Authors:** Mubbshir Khan, Haroon Yousaf, Muhammad Naqqash, Bilal Ahmad, Atizaz Ali Jan, Maria Ahmad, Asif Afridi

**Affiliations:** 1 Trauma and Orthopaedics, Queen Elizabeth Hospital Birmingham, Birmingham, GBR; 2 Orthopaedics, Mardan Medical Complex, Mardan, PAK; 3 Trauma and Orthopaedics, University Hospitals Birmingham NHS Foundation Trust, Birmingham, GBR; 4 Trauma and Orthopaedics, Hayatabad Medical Complex Peshawar, Peshawar, PAK; 5 General Surgery, Saidu Group of Teaching Hospitals, Swat, PAK; 6 Trauma and Orthopaedics, University Hospital Crosshouse, Kilmarnock, GBR; 7 Orthopaedics, Saidu Group of Teaching Hospitals, Swat, PAK; 8 Diagnostic Radiology, Mardan Medical Complex, Mardan, PAK; 9 General Surgery, Khyber Teaching Hospital, Peshawar, PAK; 10 Trauma and Orthopaedics, University Hospitals Birmingham, Birmingham, GBR

**Keywords:** avascular necrosis, core decompression, fibular strut graft, hip surgery, postoperative pain

## Abstract

Introduction: There is limited local data comparing the outcomes of core decompression and fibular strut grafting in early-stage avascular necrosis (AVN) of the hip. International findings may not be directly applicable due to population-specific factors such as genetics, lifestyle, and healthcare access. Early identification of the optimal surgical option is essential for improving patient counseling, postoperative recovery, and overall clinical outcomes.

Objective: To compare postoperative pain and functional outcomes of core decompression versus fibular strut grafting in patients with early-stage AVN of the hip.

Methodology: This prospective, randomized comparative cohort study was conducted at the Department of Orthopedic Surgery, Khyber Teaching Hospital, Peshawar, from 14th March 2023 to 13th September 2023. A total of 112 patients aged 18-50 years with Ficat and Arlet stage I-II AVN were enrolled and equally allocated into the core decompression or fibular strut graft groups using blocked randomization. Postoperative pain was assessed at 12 weeks using the visual analog scale (VAS), with a score >3 considered significant. Functional status was evaluated using the Hip Disability and Osteoarthritis Outcome Score (HOOS). Data were analyzed using chi-square tests for categorical variables and appropriate tests for functional scores, with p ≤ 0.05 considered statistically significant.

Results: Significant postoperative pain (VAS > 3) was observed in 11 (19.6%) patients in the core decompression group and 17 (30.4%) in the fibular strut graft group (χ² = 1.708, p = 0.190). Stratification by age, gender, and BMI consistently showed lower pain rates in the core decompression group, though differences were not statistically significant. HOOS functional scores demonstrated a similar trend favoring core decompression, but these differences also did not reach statistical significance.

Conclusion: Core decompression was associated with fewer patients experiencing significant postoperative pain and showed a trend toward better short-term functional outcomes compared to fibular strut grafting. Its minimally invasive nature may make it a preferable option for early pain management in patients with early-stage AVN of the hip. However, the lack of statistical significance and the short follow-up period warrant further studies with larger sample sizes and inclusion of long-term functional and radiological outcomes before definitive clinical recommendations can be made.

## Introduction

Avascular necrosis (AVN) of the femoral head, also referred to as osteonecrosis, is a progressive and often debilitating condition characterized by the death of bone tissue due to disruption of its blood supply [1]. This pathological process frequently leads to the collapse of the femoral head and eventual degenerative arthritis of the hip joint [2]. AVN is a notable cause of hip disability in young adults, accounting for approximately 5% to 12% of total hip arthroplasties worldwide [3]. The condition predominantly affects individuals under 40 years of age, a group in whom joint replacement surgeries are challenging due to their higher activity levels and longer life expectancy, often necessitating multiple revision procedures over a lifetime [4].

The etiology of AVN is multifactorial, encompassing both traumatic and non-traumatic causes. Traumatic factors include femoral neck fractures and hip dislocations, which can directly compromise the vascular supply to the femoral head [5]. Non-traumatic causes are varied and may involve prolonged corticosteroid use, alcohol misuse, coagulation disorders, sickle cell disease, autoimmune conditions, and idiopathic origins where no cause is identifiable [6]. The pathophysiology involves vascular impairment leading to hypoxia, osteocyte apoptosis, subchondral bone collapse, and eventual joint degeneration [7]. Without timely intervention, the disease progresses relentlessly, resulting in significant functional impairment and pain [8].

Management strategies for AVN vary according to disease stage, patient age, and lesion size. In the early stages, joint-preserving procedures aim to delay or prevent femoral head collapse, thereby postponing the need for total hip arthroplasty. [9] Among these, core decompression is one of the most widely used surgical techniques. This procedure involves drilling into the necrotic area to reduce intraosseous pressure, improve vascularization, and stimulate healing [10]. Its simplicity and minimally invasive nature make it attractive; however, its reported success rates vary, with some studies showing favorable outcomes and others highlighting limited efficacy [11].

An alternative surgical approach for early-stage AVN of the hip is fibular strut grafting, which offers both mechanical support and biological repair. This technique involves harvesting a segment of the fibula and inserting it into the decompressed tract of the femoral head. The graft provides structural stability to the weakened bone and promotes revascularization and bone regeneration. The key potential benefit of this method is a reduced risk of femoral head collapse compared to core decompression alone. However, fibular strut grafting is more invasive and may be associated with donor site morbidity, including pain or functional limitations at the harvest site [12,13].

Although both core decompression and fibular strut grafting have been studied internationally, their comparative effectiveness remains inconclusive, and there is a lack of direct comparative data from local patient populations. Differences in demographics, disease characteristics, and healthcare systems mean that international findings may not fully apply to our setting [14]. Addressing this gap, our study aims to compare these two procedures, focusing primarily on postoperative pain outcomes in early-stage AVN patients treated locally. Additionally, we recorded secondary outcomes such as complications and donor site morbidity to offer a more comprehensive safety profile.

## Materials and methods

Study design and setting

This study was designed as a prospective comparative cohort study conducted at the Department of Orthopedic Surgery, Medical Teaching Institution (MTI)/Khyber Teaching Hospital, Peshawar, from March 14 to September 13, 2023. The primary objective was to compare the clinical outcomes of core decompression versus fibular strut grafting in patients with early-stage AVN of the hip, focusing on postoperative pain relief and complication rates.

Sample size calculation

The sample size was determined using the WHO sample size calculator for comparing two proportions [15]. Based on prior literature, the incidence of postoperative pain after core decompression was estimated at 45.5% [16], while fibular strut grafting was associated with a lower rate of 23.2% [17]. To detect this difference with 80% statistical power and a 5% level of significance, 112 patients (56 per group) were required. Although this sample size meets statistical requirements, it approaches the minimal threshold, and this limitation is addressed in the discussion.

Inclusion and exclusion criteria

Eligible participants were adults aged 18 to 50 years, of any gender, diagnosed with stage I or II AVN of the hip based on the Ficat and Arlet classification system [18,19]. Patients presenting with local site infection, traumatic AVN etiology, psychiatric illness impairing consent or compliance, or significant comorbidities such as uncontrolled diabetes mellitus and severe cardiovascular disease were excluded to minimize confounding factors and ensure patient safety.

Patient recruitment and consent

Patients presenting to the orthopedic outpatient clinic and meeting the inclusion criteria were approached for participation. The study purpose, procedures, risks, and benefits were thoroughly explained to each patient. Written informed consent was obtained prior to enrollment. The study protocol was approved by the Institutional Review Board (IRB) of Khyber Medical College Peshawar (Approval No.: 596/DME/KMC).

Randomization and allocation concealment

Participants were randomly allocated to either the core decompression or fibular strut graft group using computer-generated blocked randomization with variable block sizes to ensure balanced group sizes and prevent allocation predictability. Allocation concealment was maintained through sequentially numbered, sealed opaque envelopes that were opened only at the time of surgery scheduling, thereby reducing selection bias.

Blinding

While blinding of surgeons was not feasible due to the nature of the surgical interventions, outcome assessors and data analysts remained blinded to group assignments throughout the study period to minimize assessment and analytical bias.

Preoperative assessment

All patients underwent a comprehensive baseline evaluation, including a detailed history of demographic data, symptom duration, steroid use, smoking, alcohol consumption, and any history of coagulation disorders. Pain was assessed using the visual analog scale (VAS) [19], and functional status was measured by the Hip Disability and Osteoarthritis Outcome Score (HOOS) [20]. Physical examination included gait analysis, hip range of motion, and assessment of local signs. Baseline laboratory investigations comprised complete blood count, liver and renal function tests, random blood sugar, and coagulation profile. Imaging studies included standard anteroposterior and frog-leg lateral pelvis radiographs, with MRI confirming AVN staging.

Surgical procedures

In the core decompression group, patients underwent surgery under spinal anesthesia with the patient positioned supine on a fracture table. Under fluoroscopic guidance, a lateral incision was made over the femoral cortex, and the fascia lata and vastus lateralis muscle fibers were carefully split to access the femur. A guidewire was inserted into the necrotic lesion, followed by advancement of an 8-mm triple reamer over the guidewire to decompress the necrotic bone. Necrotic tissue was curetted and sent for histopathological examination.

In the fibular strut graft group, after performing the core decompression steps as described above, an autologous fibular graft was harvested subperiosteally from the ipsilateral leg. The graft was shaped and drilled with multiple holes to facilitate revascularization, then inserted through the decompression tract and positioned approximately 5 mm beneath the subchondral bone to provide mechanical support.

Postoperative management and follow-up

Postoperatively, patients were instructed to maintain strict non-weight-bearing on the affected limb. Continuous 2 kg traction was applied for one week and then nocturnally for an additional two weeks. Assisted range of motion exercises were initiated after one week. Follow-up visits were scheduled at one, two, four, and 12 weeks post surgery. At each visit, VAS and HOOS scores were recorded by blinded assessors. Serial radiographs were obtained to evaluate femoral head integrity and detect any progression to collapse. MRI was repeated at 12 weeks to assess revascularization and joint preservation.

Complication monitoring

Intraoperative and postoperative complications were systematically recorded throughout the study. These included intraoperative fractures, neurovascular injury, postoperative infections, and donor site morbidity such as pain, weakness, or instability in the fibular graft group, along with any other adverse events.

Data collection and statistical analysis

Data were securely recorded and analyzed using SPSS version 22.0 (IBM Corp., Armonk, NY). Continuous variables, including age, BMI, and disease duration, were expressed as means with standard deviations, while categorical variables, such as gender and postoperative pain status, were reported as frequencies and percentages. The primary outcome, i.e., postoperative pain, was compared between groups using chi-square tests, with statistical significance set at p ≤ 0.05. Further stratified analyses were performed based on age, gender, and BMI to assess potential effect modification.

Measures to minimize bias and errors

To minimize selection bias, blocked randomization with allocation concealment was employed. Outcome assessors and data analysts were blinded to group allocation to reduce assessment and analytical bias. Surgical procedures were standardized and performed by experienced surgeons to limit procedural variability. Postoperative care protocols were uniform across groups to ensure consistent patient management. Comprehensive complication monitoring allowed for balanced evaluation of the risks and benefits associated with each intervention. Stringent inclusion and exclusion criteria were applied to control confounding variables and enhance internal validity.

## Results

In this study, 112 patients were enrolled, with 56 in each treatment group. The mean age in the core decompression group was 30.04 ± 4.014 years, while in the fibular strut graft group, it was 31.04 ± 4.323 years. As shown in Table 1, patients aged ≤ 30 years accounted for 22 (39.3%) in the core decompression group and 19 (33.9%) in the fibular strut graft group, whereas those aged > 30 years accounted for 34 (60.7%) and 37 (66.1%), respectively (χ² = 0.365, p = 0.546). Males represented 27 (48.2%) of the core decompression group and 25 (44.6%) of the fibular strut graft group, while females comprised 29 (51.8%) and 31 (55.4%), respectively (χ² = 0.132, p = 0.717). BMI ≥ 20 kg/m² was observed in 21 (37.5%) of core decompression patients and 19 (33.9%) of fibular strut graft patients (χ² = 0.144, p = 0.704). Disease duration ≤ six months was reported in 32 (57.1%) patients in the core decompression group and 27 (48.2%) in the fibular strut graft group, while > six months duration was seen in 24 (42.9%) and 29 (51.8%) patients, respectively (χ² = 0.857, p = 0.355).

**Table 1:** Baseline characteristics of the study population. Values are expressed as mean ± SD for continuous variables and n (%) for categorical variables. χ² = chi-square test; p ≤ 0.05 considered significant.

Variable	Core decompression (n=56)	Fibular strut graft (n = 56)	χ² value	p-value
Mean age (years)	30.04 ± 4.014	31.04 ± 4.323		
Age ≤ 30 years	22 (39.3%)	19 (33.9%)	0.365	0.546
Age > 30 years	34 (60.7%)	37 (66.1%)		
Gender				
Male	27 (48.2%)	25 (44.6%)	0.132	0.717
Female	29 (51.8%)	31 (55.4%)		
BMI				
≥20 kg/m²	21 (37.5%)	19 (33.9%)	0.144	0.704
<20 kg/m²	35 (62.5%)	37 (66.1%)		
Disease duration				
≤6 months	32 (57.1%)	27 (48.2%)	0.857	0.355
>6 months	24 (42.9%)	29 (51.8%)		

At 12 weeks postoperatively, significant pain (VAS > 3) was observed in 11 (19.6%) patients in the core decompression group compared to 17 (30.4%) patients in the fibular strut graft group. As shown in Table 2, the proportion of patients without significant pain was higher in the core decompression group (45, 80.4%) compared to 39 (69.6%) in the fibular strut graft group. The difference between groups was not statistically significant (χ² = 1.708, p = 0.190). Although the p-value did not meet the threshold for significance, numerically fewer patients reported significant pain in the core decompression group. These results suggest a potential clinical trend favoring core decompression for short-term pain relief. The baseline comparability of both groups supports the reliability of these findings.

**Table 2:** Primary outcome – postoperative pain (VAS > 3) at 12 weeks. Significant pain is defined as a visual analog scale (VAS) score > 3. χ² = chi-square test.

Postoperative pain	Core decompression (n = 56)	Fibular strut graft (n = 56)	χ² value	p-value
Yes (VAS > 3)	11 (19.6%)	17 (30.4%)	1.708	0.190
No (VAS ≤ 3)	45 (80.4%)	39 (69.6%)		

No major intraoperative complications were observed in either group. Minor donor site morbidity in the fibular strut graft group was limited to mild, transient pain at the graft harvest site, which resolved spontaneously without intervention. There were no cases of infection, neurovascular injury, or fracture related to the procedures. These findings support the relative safety of both techniques in the short term.

In patients aged ≤ 30 years, significant pain was observed in five (22.7%) patients of the core decompression group and eight (42.1%) patients of the fibular strut graft group. As shown in Table 3, patients without significant pain in this age group accounted for 17 (77.3%) in core decompression and 11 (57.9%) in fibular strut graft. The difference was not statistically significant (χ² = 1.741, p = 0.187). In patients aged > 30 years, significant pain occurred in six (17.6%) patients of the core decompression group and nine (24.3%) of the fibular strut graft group, with 28 (82.4%) and 28 (75.7%), respectively, reporting no significant pain. This difference also failed to reach statistical significance (χ² = 0.549, p = 0.459). Across both age strata, the numerical trend favored core decompression for fewer cases of significant postoperative pain.

**Table 3:** Stratification of postoperative pain by age. Chi-square test for difference in proportions between groups within each age stratum.

Age group	Pain, yes – core, n (%)	Pain, no – core, n (%)	Pain, yes – fibular, n (%)	Pain, no – fibular, n (%)	χ² value	p-value
≤30 years	5 (22.7%)	17 (77.3%)	8 (42.1%)	11 (57.9%)	1.741	0.187
>30 years	6 (17.6%)	28 (82.4%)	9 (24.3%)	28 (75.7%)	0.549	0.459

At 12 weeks postoperatively, patients in the core decompression group reported significantly better pain outcomes compared to those receiving fibular strut grafts (85.4 ± 10.2 vs. 79.8 ± 11.5, p = 0.045). Although other HOOS subscales, including symptoms, activities of daily living, sport and recreation, and quality of life, showed numerically higher scores in the core decompression group, these differences were not statistically significant. These results suggest that core decompression may provide superior short-term pain relief in early-stage AVN of the hip (Table 4).

**Table 4:** Comparison of HOOS subscale scores at 12 weeks postoperatively between the core decompression and fibular strut graft groups. Values are expressed as mean ± standard deviation. The Hip Disability and Osteoarthritis Outcome Score (HOOS) subscales assess patient-reported outcomes related to pain, symptoms, activities of daily living, sport/recreation function, and quality of life. * The core decompression group showed significantly better pain scores compared to the fibular strut graft group (p = 0.045). Other subscales demonstrated numerical trends favoring core decompression but did not reach statistical significance.

HOOS subscale	Core decompression (mean ± SD)	Fibular strut graft (mean ± SD)	p-value
Pain	85.4 ± 10.2	79.8 ± 11.5	0.045*
Symptoms	80.1 ± 12.0	75.5 ± 13.2	0.12
Activities of daily living	82.3 ± 9.5	78.0 ± 10.7	0.08
Sport and recreation	70.0 ± 15.3	65.2 ± 16.0	0.15
Quality of life	75.5 ± 13.7	70.1 ± 14.4	0.1

**Figure 1:**
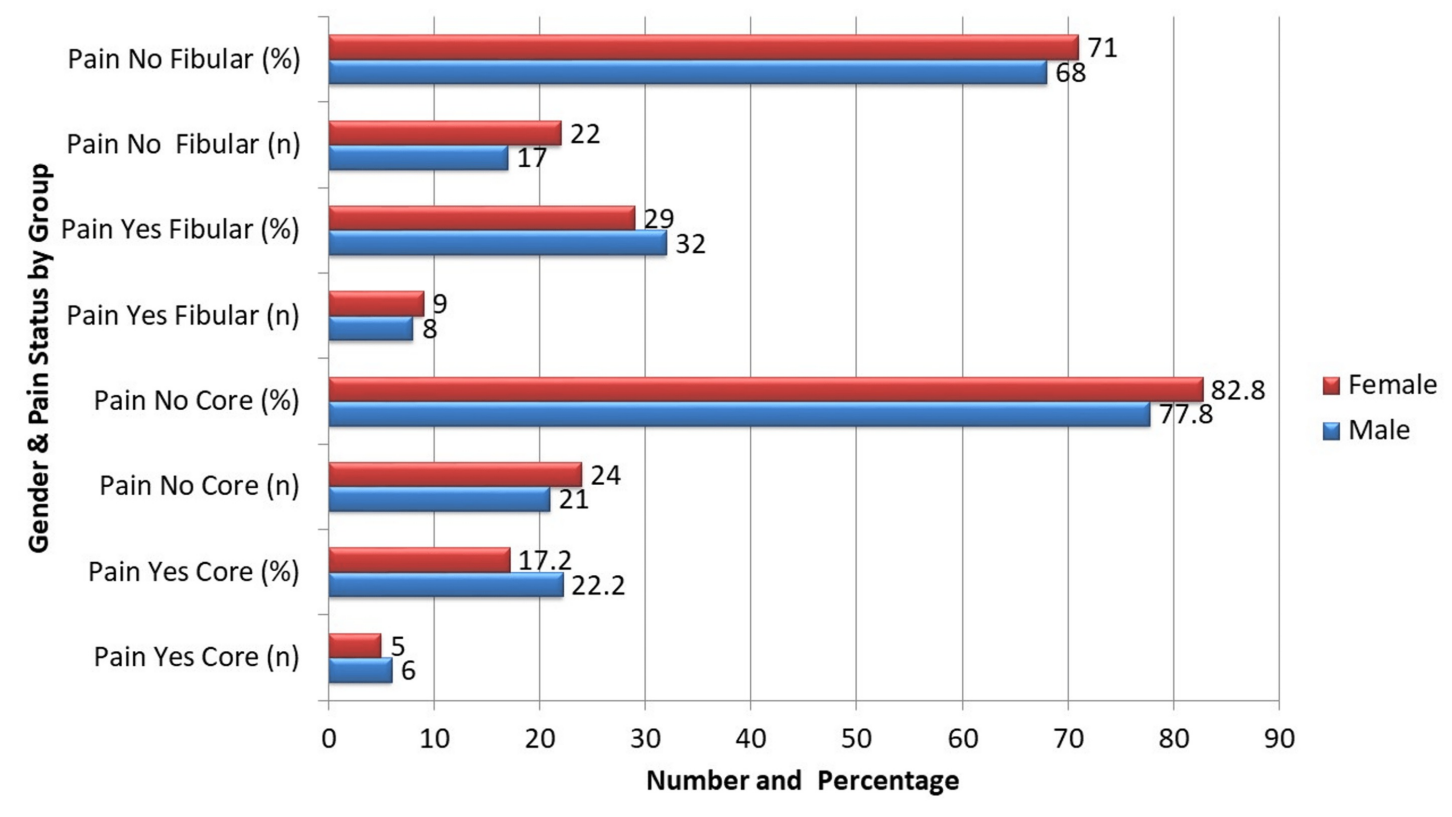
Stratification of postoperative pain by gender. χ² = chi-square test for difference in proportions between groups within each gender stratum. P-value: female = 0.32; male = 0.45. χ²: female = 0.98; male = 0.56.

As shown in Figure 1, among male patients, significant pain was found in six (22.2%) patients of the core decompression group compared to eight (32.0%) in the fibular strut graft group. No significant pain was reported in 21 (77.8%) and 17 (68.0%) patients, respectively. This difference was not statistically significant (χ² = 0.560, p = 0.454). Among female patients, significant pain occurred in five (17.2%) patients in the core decompression group and nine (29.0%) in the fibular strut graft group, with no significant pain in 24 (82.8%) and 22 (71.0%) patients, respectively. This comparison also did not reach statistical significance (χ² = 0.981, p = 0.322). In both genders, a numerically lower proportion of significant pain cases was observed in the core decompression group.

**Figure 2:**
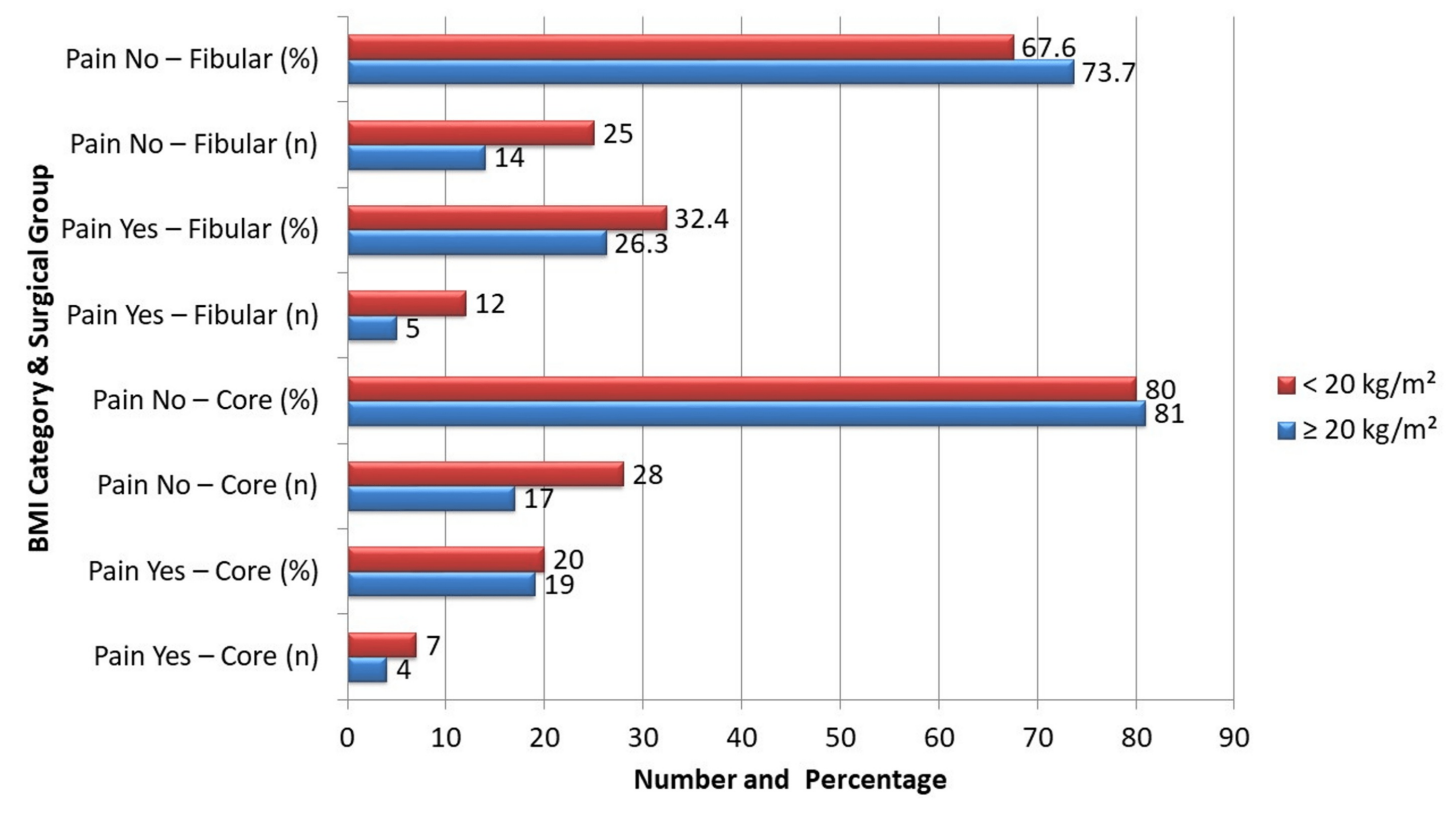
Stratification of postoperative pain by BMI. χ² = chi-square test for difference in proportions between groups within each BMI stratum. Chi-square values = 1.499 and 0.278. P-values = 0.221 and 0.598.

As shown in Figure 2, in patients with BMI ≥ 20 kg/m², significant pain was recorded in four (19.0%) patients of the core decompression group compared to five (26.3%) of the fibular strut graft group, with no significant pain in 17 (81.0%) and 14 (73.7%) patients, respectively. This difference was not statistically significant (χ² = 0.278, p = 0.598). In the BMI < 20 kg/m² category, significant pain occurred in seven (20.0%) patients of the core decompression group and 12 (32.4%) of the fibular strut graft group, with 28 (80.0%) and 25 (67.6%) patients, respectively, having no significant pain. This difference also failed to reach statistical significance (χ² = 1.499, p = 0.221). Across BMI strata, fewer patients in the core decompression group reported significant pain, consistent with the overall study trend.

## Discussion

Both core decompression and fibular strut grafting demonstrated effectiveness in managing early-stage AVN of the hip. The primary outcome of interest, i.e., significant postoperative pain defined as a VAS score greater than 3, was observed less frequently in the core decompression group (11 patients, 19.6%) compared to the fibular strut graft group (17 patients, 30.4%) at the 12-week follow-up. Although this difference did not achieve statistical significance (p = 0.190), the numerical trend favored core decompression. This finding supports the study hypothesis that core decompression may offer superior short-term pain relief in early AVN. Importantly, the trend remained consistent across various subgroups stratified by age, gender, and BMI, underscoring the robustness of the observed effect.

Comparing these results with existing literature reveals concordance with previous studies highlighting the benefits of core decompression in early AVN. Core decompression is believed to alleviate symptoms by reducing intraosseous pressure and facilitating revascularization of the affected femoral head, mechanisms that contribute to pain reduction and potentially halt disease progression [21]. International clinical trials and cohort studies have reported similar outcomes, noting that core decompression provides pain relief comparable to or better than other surgical interventions, particularly when performed in the initial stages of the disease [22]. These findings reinforce the rationale for using core decompression as an early intervention to preserve hip joint integrity while minimizing patient morbidity.

Fibular strut grafting, by contrast, serves a dual purpose: it provides mechanical structural support to the necrotic area and fills the cavitary defect left after debridement of necrotic bone. However, this procedure is more invasive, often requiring longer operative times and more extensive soft tissue dissection. Such factors may contribute to increased early postoperative discomfort, as reflected in the higher proportion of patients reporting significant pain in the grafting group [23]. The invasiveness of fibular grafting may also be associated with a longer recovery period, which could affect short-term patient-reported outcomes. Nonetheless, it remains a valuable technique, especially in cases where mechanical support is critical to preventing femoral head collapse.

The findings of this study align well with previously published data supporting the use of core decompression as a first-line surgical option in early AVN, particularly when the clinical priority is to achieve prompt pain relief with a less invasive approach [24]. While both techniques can improve patient outcomes, the less invasive nature of core decompression combined with its favorable pain profile in the short term may offer important advantages in clinical decision-making. Further long-term studies with larger sample sizes are needed to clarify the comparative effectiveness of these procedures on functional recovery and radiographic progression.

Limitations and future suggestions

Limitations of this study include its single-center design and the relatively short follow-up duration of 12 weeks, which may not adequately capture the progressive nature of AVN, such as femoral head collapse, functional decline, or the eventual need for arthroplasty. Furthermore, the study’s reliance on subjective pain assessment using the VAS, while widely accepted, can be influenced by individual patient perception and variability. Although blocked randomization was employed to minimize selection bias, the observational cohort design and practical constraints may limit control over confounding variables compared to a fully randomized controlled trial.

While we included the HOOS as a secondary functional outcome measure, longer-term assessment of functional improvement, radiological progression, and the need for total hip replacement were not evaluated in this study but remain critical endpoints for future research.

Future recommendations emphasize the need for multi-center prospective randomized controlled trials with larger sample sizes and longer follow-up periods to assess a broader range of outcomes, including pain, functional improvement via HOOS, radiographic changes, and joint preservation. Additionally, investigating the potential benefits of biologic augmentation techniques, such as platelet-rich plasma or stem cells combined with core decompression, could enhance treatment efficacy and warrant further exploration.

## Conclusions

In this prospective randomized comparative cohort study, core decompression was associated with a lower proportion of patients experiencing significant postoperative pain compared to fibular strut grafting in early-stage AVN of the hip, thus supporting the study hypothesis. Although the difference did not reach statistical significance, the consistent trend across age, gender, and BMI subgroups suggests a potential clinical advantage of core decompression for short-term pain relief. Given its minimally invasive nature and more favorable postoperative pain profile, core decompression may be considered the preferred surgical option for managing early-stage AVN. However, these findings need to be interpreted with caution. Further research, ideally involving larger, multi-center randomized controlled trials with extended follow-up periods, is necessary to validate these results and to evaluate long-term functional outcomes using measures such as the HOOS, radiological progression, and joint preservation.
